# Advancing regulatory science and assessment of FDA REMS programs: A mixed-methods evaluation examining physician survey response

**DOI:** 10.1017/cts.2019.400

**Published:** 2019-09-13

**Authors:** Sarah E. Brewer, Elizabeth J. Campagna, Elaine H. Morrato

**Affiliations:** 1 Adult and Child Consortium for Health Outcomes Research and Delivery Science (ACCORDS), University of Colorado Anschutz Medical Campus, Aurora, CO, USA; 2 Department of Family Medicine, School of Medicine, University of Colorado Anschutz Medical Campus, Aurora, CO, USA; 3 Health and Behavioral Sciences, University of Colorado Denver, Denver, CO, USA; 4 Department of Health Systems, Management and Policy, Colorado School of Public Health, University of Colorado Anschutz Medical Campus, Aurora, CO, USA

**Keywords:** Case illustration, methods, physician surveys, provider surveys, response rates, risk management, systematic review

## Abstract

**Purpose:**

Food and Drug Administration’s (FDA) *Draft Guidance for Industry* on pharmaceutical REMS (Risk Evaluation and Mitigation Strategies) assessment and survey methodology highlights physician knowledge–attitudes–behaviors (KAB) surveys as regulatory science tools. This mixed-methods evaluation advances regulatory science and the assessment of FDA REMS programs when using physician surveys. We: (1) reviewed published physician survey response rates; and (2) assessed response bias in a simulation study of secondary survey data using different accrual cut-off strategies.

**Methods:**

A systematic literature review was conducted of US physician surveys (2000–2014) on pharmaceutical use (*n* = 75). Kruskal–Wallis tests were used to examine the relationships between response rates and survey design characteristics. The simulation was conducted using secondary data from a population-based physician KAB survey on diabetes risk management with antipsychotic use in Missouri Medicaid (*n* = 973 accrued over 30 weeks). Survey item responses were compared using Pearson’s chi-square tests for two faster completion simulations: Fixed Sample (*n* = 300) and Fixed Time (8 weeks).

**Results:**

Survey response rates ranged from 7% to 100% (median = 48%, IQR = 34%–68%). Surveys of targeted populations and surveys using member lists were associated with higher response rates (*p* = 0.02). In the simulation, 9 of 20 (45%) KAB items, including diabetes screening advocacy, differed significantly using the smaller Fixed Sample strategy (achieved in 12 days) versus full accrual. Fewer response differences were found using the Fixed Time strategy (2 of 20 [10%] items).

**Conclusions:**

Published data on physician surveys report low response rates with most associated with the sample source selected. FDA REMS assessments should include formal evaluation of survey accrual and response bias.

## Introduction

A core responsibility of the US Food and Drug Administration (FDA) is to protect patients by applying the best science to its regulatory decision-making ranging from pre-market review of the efficacy and safety of new drugs to post-market product surveillance. Post-marketing safety evaluation is a critical pharmacovigilance activity in the benefit–risk communication and risk management life cycle for medicinal products.^1^ Modern risk management assessment includes advanced statistical methods using large population-based and government-sponsored clinical informatics networks of administrative and electronic health data and sophisticated data mining strategies to identify safety signals.^1^^,^^2^ In addition, knowledge–attitudes–behaviors (also known as KAB) surveys conducted among patients and physicians supplement epidemiologic data analysis.^3^^–^^8^ For example, survey findings are often presented at public FDA advisory committee meetings discussing issues of drug safety and risk management, for example, the Risk Evaluation and Mitigation Strategy (REMS) review for extended-release and long-acting opioid analgesics.^9^

The FDA defines regulatory science as the science of “developing new tools, standards, and approaches to assess the safety, efficacy, quality, and performance of all FDA-regulated products.”^10^ In its *Strategic Plan for Advancing Regulatory Science*, FDA identifies strengthening social and behavioral science as a key priority area.^10^ Social sciences provide the scientific framework and methods necessary for assessing the impact of product information on clinician and patient understanding and for evaluating the effectiveness of benefit–risk communication on clinical behaviors and patient health outcomes.^10^

In 2019, the FDA issued draft guidance for industry on *REMS Assessment: Planning and Reporting* and *Survey Methodologies to Assess REMS Goals that Relate to Knowledge*.^11^ The Food and Drug Administration Amendments Act of 2007 (FDAAA) and later amended by the Food and Drug Administration Safety and Innovation Act (FDASIA) authorize FDA to require REMS for certain drugs if FDA determines that an REMS is necessary to ensure that the drug’s benefits outweigh its risks when used in clinical practice. Approved products with REMS requirements can be found at REMS@FDA,^12^ and currently reflect 81 active programs (as of 5/1/19). Examples of REMS programs include: mitigating teratogenic risk for isotretinoin (a treatment for severe acne), mitigating the risk of severe neutropenia for clozapine (an antipsychotic), and mitigating the risk of abuse and misuse for opioid analgesics (required for pain management).

Drug manufacturers are required to submit assessments at specified intervals after REMS implementation.^13^ REMS assessments can evaluate the impact of the program on proximal measures (e.g., knowledge, attitudes, and behaviors) and distal measures (safety-related health outcomes) and can assess the effectiveness of individual programmatic components, such as patient labeling and packaging, communication plans for healthcare professionals, and other healthcare system elements implemented to ensure safe use of the medication. Many REMS include a goal related to physician knowledge, or the act of being aware of certain drug risks and understanding what risk-mitigation behaviors are required. In this situation, REMS assessment plans generally include a survey to evaluate patients’ and healthcare providers’ understanding of the serious risks associated with, and safe use of, the drug.

In 2012, the FDA convened a public workshop on social science methodologies and the use of surveys to assess whether an REMS is meeting its knowledge goals.^14^ Panel members discussed methodological challenges in survey administration. Issues raised included: determining a representative sampling frame, fielding rapid surveys given the regulatory review timeline, addressing low survey response rate and potential bias, and determining what level of self-reported knowledge or behavior signifies sufficient risk management success. Historical challenges with physician survey response are well documented.^15^^–^^17^ Importantly, though, prior literature examining factors affecting response rates largely pre-dates a long-standing decline in survey response rates that has accelerated in recent years^18^ and the advance of internet and other digital polling methods.

The goal for this mixed-methods evaluation is to advance regulatory science and the assessment of FDA REMS programs when using physician surveys. The first objective was to provide a more up-to-date and focused understanding of physician response rates for surveys on pharmaceutical products given various study designs. For this objective, we conducted a systematic literature review of physician surveys conducted in the USA and published over a 15-year period. Knowing that timeliness of regulatory-decision making is paramount, the second objective was to evaluate and simulate the effects of using two different survey administration strategies for determining how quickly to close a physician survey. In order to evaluate the differences in interpretations of results and conclusions from a physician KAB survey, we compared both characteristics of the respondents and their answers to survey items ascertaining risk knowledge, attitudes, and behavioral intent using different survey accrual stoppage criteria. For this objective, we conducted a secondary analysis case study simulation using source data from a population-based prescriber survey, conducted by the authors, which assessed diabetes risk management for patients receiving second-generation antipsychotics.

## Methods

### Systematic Review of Physician Surveys

A systematic review was conducted in PubMed using Medical Subject Headings (MeSH) terms and keywords to identify physician surveys published between 1/1/2000 and 12/31/2014. Articles were excluded if they did not survey US physicians, did not report survey methodology or response rates, or did not have prescription medication as the survey topic. Articles published prior to 2000 were excluded due to the lack of broadly available and reliable internet connections through which email or mixed-mode surveys are conducted, which are commonly used in health services research today.^19^^,^^20^ Surveys of US physicians on topics of pharmaceutical medications were selected to align with FDA-regulated drug risk management. In addition, articles were added which were referred by our study advisory committee of experts, and relevant citations from multiple included articles from the PubMed search were added to the pool of articles for review to ensure a broad representation of manuscripts on the topic. Appendix A provides a description of the search terms and selection process.

Among the articles identified for screening under the criteria described above, using MeSH terms (*n* = 164) and additionally referred articles (*n* = 24), an abstract review was performed to identify articles meeting eligibility criteria. Among eligible publications (*n* = 70 articles, *n* = 75 distinct surveys), full manuscript reviews were conducted to identify physician response rates and survey method factors that could influence response based on reported determinants.^17^^,^^21^ The following parameters were abstracted: survey sample data source (including size of sample), physician specialty, number of contact attempts, survey mode (mail, email, or mixed modality), incentive amounts, study design (population-based (total census) vs. random sample). Data sources were categorized as either commercially available contact lists purchased by the researchers or internal membership or contact lists available to an organization. Table 1 and Appendix B provide a listing of the publications selected for review and their survey characteristics.

**Table 1.** Response Rate by Survey Design and Method Characteristics

Survey Characteristic	Value	Number of Articles	Median Response Rate (Interquartile Range)	*p*-value*
Location	National	39	47.0 (33.0–65.0)	0.678
	State	34	48.9 (34.6–71.0)	
Sample Source	Commercially available	24	**45.1 (30.0–63.8)**	**0.018**
	Internal member lists	14	**64.7 (41.5–83.0)**	
Sampling Method	Population	27	53.0 (35.9–80.0)	0.091
	Random	34	42.8 (32.0–60.0)	
Target sample (Number Contacted)	<100	5	**77.0 (71.0–80.0)**	**0.02**
	100–199	9	**71.0 (48.0–84.0)**	
	200–499	7	**53.0 (33.0–65.0)**	
	500–999	15	**38.0 (25.5–67.0)**	
	1000–1499	15	**42.0 (34.0–66.0)**	
	1500–1999	8	**46.8 (26.2–55.2)**	
	2000+	15	**47.3 (28.4–63.2)**	
Contact Method	Email	5	32.0 (18.0–62.5)	0.061
	Mail	36	43.3 (32.0–61.6)	
	Mixed	24	55.0 (38.5–73.5)	
Contact Attempts	1	13	48.0 (22.0–60.0)	0.481
	2	9	41.0 (32.0–48.0)	
	3	20	50.5 (33.8–68.8)	
	4	9	49.5 (30.0–71.4)	
	5+	11	47.0 (38.0–72.0)	

* Kruskal–Wallis test *p* < 0.05. Highlighted in bold.

Source: *n* = 75 published reports of US physician surveys, 2000–2014.

Note: 75 published articles were reviewed. Of these, missing data on individual comparison included: 2 articles reported a location within a large health system and 1 article did not report location; 37 articles did not report their sample source; 14 articles used sample methods such as an RCT, polling, quota sample, or convenience sample and were excluded; 1 did not report number of physicians contacted; 7 contacted physicians by some other method and 3 did not report the contact method; 13 did not report the total number of contacts attempts per physician.

The Kruskal–Wallis test was used to examine bivariate relationships between response rates and each of the survey design factors and methods. The subset of data with non-missing survey design factors and methods was insufficient for a multivariable model of the response rate.

### Case Study Simulation of Different Survey Design Criteria for Study Completion

The data source for the simulation is secondary data from a state-wide population-based physician survey conducted to understand underlying mechanisms affecting drug-related cardiometabolic risk management for adults starting second-generation antipsychotic medication.^22^ The survey was one component of a mixed-methods assessment conducted by the authors to assess risk management gaps for targeted intervention.

The survey data provided a unique dataset for the case study simulation of physician survey response for two reasons. First, it included similar questions to a typical KAB survey conducted as part of an REMS program evaluation. Second, the authors had access to longitudinal participant accrual data and detailed survey administration information which permitted a statistical analysis of different survey closure thresholds on both respondent characteristics and risk knowledge and safe use risk behavior responses.

***Survey data***. The survey was fielded among all prescribers of antipsychotic medication for Missouri Medicaid beneficiaries to assess their knowledge, attitudes, and behaviors regarding diabetes and lipid screening and risk management. Cardiometabolic risk information (including drug warnings, “Dear Physician Letters,” and continuing medical education) had been widely disseminated over the past decade.^23^^,^^24^ This 20-item KAB survey included 12 knowledge and attitude items, 8 risk management behavior items, and also included a single 10-point Net Promoter item assessing the likelihood of a respondent recommending metabolic screening behaviors to a colleague.

These survey data were selected as a dataset for this secondary case study of physician survey response because it included similar questions to a typical KAB survey conducted as part of a REMS process and remained open significantly longer than a typical REMS survey, allowing our study team to evaluate the potential implications of different survey closure timelines. The physician survey received approval from the Colorado Multiple Institution Review Board (COMIRB) and was performed under specifications outlined in a Data Use Agreement with Missouri Medicaid.

The survey was administered in 2014 to the full census of physicians (*n* = 4841) who prescribed antipsychotic medication to Medicaid adult patients in Missouri in 2013. Provider identification and addresses were obtained from Missouri Medicaid administrative data and supplemented with publicly available physician market data (ProviderPRO database from Healthcare Data Solutions). In addition, survey administration followed best practices for maximizing survey response,^19^^,^^20^ and information was collected to evaluate time-to-response rates and nonresponse bias.^22^ A pre-survey letter was sent to all participants from the Director of the Department of Mental Health signifying the importance of the survey. Subsequent surveys were mailed per standardized protocols and up to three survey attempts plus one reminder were made to each provider over 6 weeks. Physicians received a $2 incentive with the initial mailed survey. An additional attempt to reach nonrespondent provider was made by phone and fax at 7 and 8 weeks. Finally, when available, email attempts were made to the physician or their office administrator to solicit a response. Survey mailing dates, all returned or undeliverable mail, active opt-outs, reported reasons for opting out, and response receipt dates were documented. To maximize response rates, there was no *a priori* cutoff date for closing the survey. The survey was effectively closed at 30 weeks when analysis began.

***Statistical analyses***. In all, 973 physicians returned a completed antipsychotic metabolic risk KAB survey (20.1% crude response) and 628 physicians actively opted out of the survey (13.0%), for a total 33.1% response rate. Previously reported responder–nonresponder analysis found that respondents had more Medicaid claims for antipsychotic prescriptions and were more likely to be community mental health or primary care providers.^22^ Commonly reported reasons for opting out included primarily treating children, prescribing too few antipsychotics, or being a dentist. Completers, opt-outs, and nonrespondents were statistically different on all available demographic characteristics.

In the current secondary analysis, the Missouri Medicaid survey provides the probability-based sample of respondents for a case study simulation comparing two accrual thresholds for survey closure used in pharmaceutical risk management assessment: Fixed Sample (survey ends when a prespecified sample (N) threshold is achieved) and Fixed Time (survey ends when a prespecified time point is reached).

The Fixed Sample group included respondents who submitted their surveys by the date the 300th survey was received by the study team (*n* = 319). This is a common approach used in public opinion polling and corresponds with a margin of error of approximately ±6 percentage points.^25^ The Fixed Time group included respondents whose surveys were received within 8 weeks of the first survey mailing (*n* = 810), representing a typical rapid-response survey time used in public health policy research.^19^

Graphing was used to visually compare differences in response over time. Pearson’s chi-square tests of association were used to determine whether different thresholds for closing the survey would have resulted in statistically different physician characteristics and survey responses. Responses for respondents who qualified for each survey accrual threshold group (e.g., first 300 respondents or respondents who completed the survey within 8 weeks) were compared to responses for respondents whose survey was returned after that accrual threshold.

## Results

### Systematic Review of Physician Surveys

We reviewed 188 abstracts and identified 70 articles and 75 distinct surveys for full manuscript reviews. Appendix A shows a CONSORT-style diagram outlining the inclusion and exclusion of articles. Of note in our reviews was the amount of information about physician surveys that was not reported in the manuscripts. Of the 75 surveys reviewed, missing data on individual comparison included: 2 articles reported a location within a large health system and 1 article did not report location; 37 articles did not report their sample source; 1 did not report number of physicians contacted; 7 contacted physicians by some other method and 3 did not report the contact method; 13 did not report the total number of contacts attempts per physician.

Table 1 presents median response rate by survey design characteristics and methods. The mean reported physician survey response rate in the published literature was 51% (*SD* = 22%). Reported response rates ranged from 7% to 100% (median = 48%, IQR = 34%–68%). Response rates were lower when a larger sample was contacted (*p* = 0.02). Population-based samples trended toward higher response rates than random samples (53% vs. 43%, *p* = 0.091). Mixed-mode contact methods yielded higher response rates than mailed or emailed surveys alone (55% vs. 43% vs. 32%, *p* = 0.061). There were no differences in response rate by whether the sample was nationwide or statewide/local or number of contact attempts made.

**Fig. 1.**
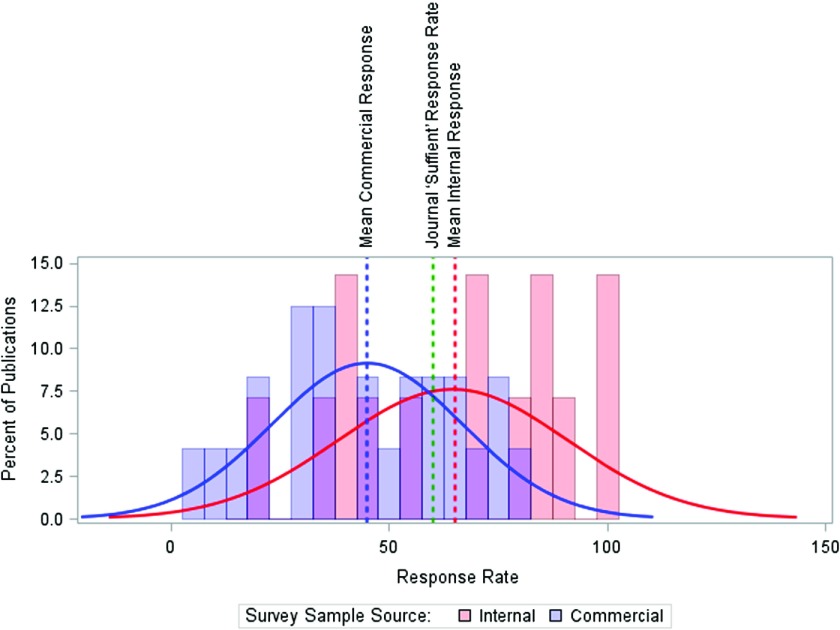
Response Rate by Survey Sample Source. Note: The median response times are slightly higher than the mean: 47% for commercially-available sources, 71% for non-commercially available internal member lists. Source: *n* = 75 published reports of US physician surveys, 2000–2014.

Figure 1 shows the differences in response rate by survey sample source. Commercially-available sample lists had lower median response rate when compared to surveys administered to physicians from internal organizational lists (45% vs. 65%, *p* = 0.02); however, 37 of 75 (49%) surveys did not report their sample source. The figure provides context for publication requirements, by comparing these two means against a threshold response rate of 60% which the *JAMA Network* defines as a sufficient response.^26^ The *JAMA Network* threshold was selected for the large number of journals in this network and high average impact of the network’s journals. In addition, we found few other journals publicly instruct authors on their sufficient response requirements, while the *JAMA Network* makes these clear in their author instructions.

Data reported on financial incentives was too sparse to review; only 18 of 75 surveys reported any type of incentive. No publications reported a survey response bias analysis.

### Case Study Simulation Simulating Different Survey Cutoff Criteria

**Fig. 2.**
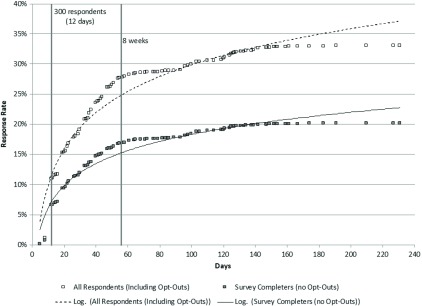
Survey response rate over time (*n* = 4823). Note: Respondents with no survey return date are not shown, *n* = 18.

Figure 2 shows the survey response curve over time for completed surveys and for all responses (completed surveys + respondents actively opting out of the survey and returned the survey screener). The time required to reach 300 responders was 12 days after the first survey mailing. The response curve approximated a log-linear curve. The response rate continued to raise over the first 8 weeks and plateaued after the 8-week mark, with a small incremental increase (approximately 3% points) in returned responses following successive attempts to reach respondents during the 90–120 day time period and maximize response.

Table 2 describes survey respondent characteristics. In the Fixed Sample group (*n* = 319), respondents were 70.1% male, majority (52.2%) born in 1946–1964, and 83.4% practiced in Missouri. This group of very early survey respondents (within 12 days) had a statistically different physician specialty profile (i.e., more likely to be a primary care provider versus behavioral health provider) (χ^2^ = 12.155, *df* =5; *p* = 0.033) and practice in different clinical setting (i.e., less likely to practice in a Community Mental Health Clinic) (χ^2^ = 34.343, *df* = 1; *p* < 0.001) compared to later respondents. The earlier survey respondents were also more likely to treat fewer patients taking antipsychotics in a typical work week than later responders (χ^2^ = 10.380, *df* = 4; *p* = 0.034).

**Table 2.** Case illustration: Provider Characteristics by Response Time

Characteristic	Response	Fixed Sample				Fixed Time				Probability Sample
		First 300 Respondents				First 8 Weeks				All Respondents
		*n* = 319				*n* = 810				*n* = 973
		% (*n*)	χ^2^	*df*	*p*	% (*n*)	χ^2^	*df*	*p*	% (*n*)
**Demographic**										
What is your gender?	Male	70.1% (223)	3.74	1	0.05	65.8% (532)	0.01	1	0.92	65.9% (640)
	Female	29.9% (95)				34.2% (276)				34.1% (331)
In what year were you born?	1901–1945	11.6% (37)	0.07	2	0.96	10.8% (87)	2.28	2	0.32	11.2% (109)
	1946–1964	52.2% (166)				53.3% (430)				52.3% (507)
	1965–2000	36.2% (115)				35.9% (289)				36.4% (353)
CMHC Prescriber in CY 2013^ab^	No	**96.9% (309)**	**34.34**	1	**0.00**	**87.2% (706)**	**4.83**	1	**0.03**	88.2% (858)
	Yes	**3.1% (10)**				**12.8% (104)**				11.8% (115)
Provider Specialty^a^	Primary Care	**63.9% (204)**	**12.16**	5	**0.03**	56.3% (456)	6.69	5	0.24	57.9% (563)
	Behavioral Health	**23.5% (75)**				31.1% (252)				30.0% (292)
	Emergency Medicine	**2.8% (9)**				2.7% (22)				2.5% (24)
	Neurology	**2.2% (7)**				2.8% (23)				3.0% (29)
	Other	**7.2% (23)**				6.5% (53)				6.2% (60)
	Unknown	**0.3% (1)**				0.5% (4)				0.5% (5)
Number of new starts of oral antipsychotics	0	15.4% (49)	2.60	4	0.63	15.6% (126)	8.62	4	0.07	14.3% (139)
	1–2	16.9% (54)				15.9% (129)				15.9% (155)
	3–11	19.4% (62)				18.5% (150)				18.5% (180)
	12 or more	48.3% (154)				50.0% (405)				51.3% (499)
State	Missouri	83.4% (266)	3.23	1	0.07	85.4% (692)	2.58	1	0.11	86.2% (839)
	Border State	16.6% (53)				14.6% (118)				13.8% (134)
Urban Setting	No	37.6% (120)	1.16	1	0.28	36.0% (292)	1.35	1	0.25	35.3% (343)
	Yes	62.4% (199)				64.0% (518)				64.7% (630)
**Clinical Practice**										
In a typical work week, how many outpatient visits do you provide?	None	2.0% (6)	5.88	3	0.12	2.5% (19)	4.17	3	0.24	2.3% (21)
	1–49	21.3% (64)				23.3% (176)				23.3% (211)
	50–99	40.7% (122)				42.3% (319)				43.6% (394)
	100+	36.0% (108)				31.8% (240)				30.8% (278)
Of those, what percent are adults?	None	3.7% (11)	0.60	4	0.96	4.1% (31)	1.68	4	0.80	3.9% (35)
	1%–49%	7.7% (23)				7.5% (56)				7.3% (66)
	50%–79%	19.1% (57)				18.3% (137)				18.2% (164)
	80%–99%	34.8% (104)				33.5% (251)				34.3% (308)
	100%	34.8% (104)				36.6% (274)				36.3% (326)
In a typical work week, what percent of your adult patients are taking antipsychotics?^a^ ^b^	None	**2.0% (6)**	**10.38**	4	**0.03**	**3.0% (22)**	**13.20**	4	**0.01**	2.9% (26)
	1%–9%	**40.0% (118)**				**35.9% (265)**				38.0% (336)
	10%–24%	**32.2% (95)**				**29.3% (216)**				27.7% (245)
	25%–49%	**11.9% (35)**				**12.2% (90)**				12.8% (113)
	50%–100%	**13.9% (41)**				**19.6% (145)**				18.6% (164)
Of those, what percent have you personally prescribed?^b^	None	19.2% (56)	1.81	4	0.77	**18.9% (138)**	**10.35**	4	**0.04**	17.6% (154)
	1%–9%	29.9% (87)				**27.4% (200)**				29.0% (254)
	10%–49%	18.9% (55)				**19.3% (141)**				18.7% (164)
	50%–99%	19.2% (56)				**21.8% (159)**				21.4% (187)
	100%	12.7% (37)				**12.7% (93)**				13.3% (116)

Notes: Data from a knowledge–attitudes–behaviors survey of Medicaid providers in Missouri who prescribed atypical antipsychotic medication in 2013.The first 300 surveys were received by day 12. Missing responses (present for sex and age) have been excluded (<1%). Border states included: Arkansas, Illinois, Iowa, Kansas, and Tennessee. CMHC – Community Mental Health Center

a Pearson’s chi-square test of association *p* < 0.05 when comparing survey item response among respondents in the First 300 Respondents group vs. later. Highlighted in bold.

b Pearson’s chi-square test of association *p* < 0.05 when comparing survey item response among respondents returning surveys within 8 weeks vs. later. Highlighted in bold.

In the Fixed Time group (*n* = 810), respondents were 65.8% male, 53.3% born in 1946–1964, and 85.4% practiced in Missouri. Unlike the very early respondents, the group of survey respondents within 8 weeks did not differ from later respondents on physician specialty profile (χ^2^ = 6.694, *df* = 5; *p* = 0.224). This Fixed Time sample was more likely than later respondents to work in a community mental health center (χ^2^ = 4.830, *df* = 1; *p* = 0.028). Physicians responding within 8 weeks were more likely to have a greater percent of patients taking antipsychotics in a typical work week (χ^2^ = 13.204, *df* = 4; *p* = 0.010) and differed in the percentage of patients for whom they had personally prescribed the antipsychotics than in later responders (χ^2^ = 10.349, *df* = 4; *p* = 0.035).

Table 3 presents the KAB survey responses by survey accrual threshold group. Providers making the Fixed Sample cut-off (300 respondents, 12 days) had more varied responses than providers responding later, while the Fixed Time cut-off (8-weeks) group had fewer differences from the providers who responded after 8 weeks. On 9 of the 20 (45%) KAB items, the Fixed Sample group differed from later respondents. When comparing the 8-week cut off group to later respondents, there were significant differences for only 2 out of these 20 items (10%). For example, they were less likely to strongly agree that all adults starting antipsychotics should receive metabolic testing, and that their patients taking antipsychotics were at high risk for diabetes. Their self-reported behavioral intent to order glucose and lipid testing was also consistently lower. In addition, the Fixed Sample group was significantly different from later responders on the Net Promoter score, while the Fixed Time group did not differ from later respondents. This indicates that the earliest respondents were less likely to advocate for metabolic screening of adults receiving antipsychotics compared to later respondent groups.

**Table 3.** Physician-Reported Screening Attitudes by Response Time

Survey Question		Response	Fixed Sample	Fixed Time	Probability Sample
			First 300 Respondents	First 8 Weeks	All Respondents
			*n* = 319	*n* = 810	*n* = 973
**Knowledge**	All adults starting antipsychotic drugs should have a baseline glucose and lipid test.^a^	Agree Strongly	**47.6% (148)**	54.8% (431)	55.2% (521)
	My patients are at high risk for diabetes.^a^	Agree Strongly	**35.6% (109)**	40.8% (314)	40.5% (374)
	Interpreting blood glucose values and diagnosing diabetes.	Very Confident	81.6% (258)	79.6% (636)	80.3% (769)
**Attitudes**	My practice is responsible for glucose and lipid screening.	Agree Strongly	69.4% (218)	69.1% (549)	70.2% (667)
	Patients forget to get lab work.	Agree Strongly	23.2% (72)	23.5% (185)	23.5% (221)
	Fasting makes it difficult for my patients to comply.	Agree Strongly	9.7% (30)	10.7% (84)	10.2% (96)
	The added time or needed transportation is inconvenient for patients.	Agree Strongly	10.4% (32)	11.1% (87)	10.6% (99)
	Patients do not see screening as a priority.^b^	Agree Strongly	21.5% (67)	**19.5% (154)**	18.0% (170)
	I do not have the necessary equipment at my office/clinic.	Agree Strongly	14.7% (46)	16.9% (133)	16.2% (152)
	I have difficulty getting the lab results if done elsewhere.	Agree Strongly	9.9% (31)	12.2% (96)	12.3% (116)
	Screening adds complexity to my clinical workload.	Agree Strongly	9.9% (31)	9.4% (74)	9.0% (85)
	At this time, metabolic screening is not a priority for me or my organization.	Agree Strongly	3.2% (10)	3.3% (26)	3.4% (32)
**Behaviors**	At antipsychotic initiation:				
	Order a blood glucose test? (fasting or A1C)^a^	Definitely	**35.0% (109)**	40.5% (320)	41.2% (391)
	Order a lipids profile?^a^	Definitely	**33.4% (104)**	38.6% (304)	39.0% (368)
	At 1-year follow-up:				
	Order a blood glucose test? (fasting or A1C)^a^	Definitely	**53.7% (167)**	58.6% (462)	59.7% (565)
	Order a lipids profile?^a^	Definitely	**51.3% (159)**	56.1% (440)	57.2% (539)
	When there is an abnormal glucose value:				
	Order a confirmatory blood glucose test?^a^ ^,^ ^b^	Definitely	**40.7% (123)**	**44.5% (340)**	46.5% (426)
	Prescribe anti-diabetic medication?	Definitely	13.7% (41)	12.1% (92)	12.6% (114)
	Refer the patient to a primary care physician?^a^	Definitely	**32.7% (97)**	40.3% (307)	39.4% (358)
	Record and carefully monitor?^a^	Definitely	**65.2% (199)**	69.3% (536)	69.7% (648)
	Metabolic screening advocacy Net Promoter Score^a^ *	Detractor	**34.0% (105)**	29.7% (235)	28.6% (272)
		Neutral	**21.0% (65)**	19.2% (152)	18.9% (180)
		Promoter	**45.0% (139)**	51.1% (405)	52.5% (499)

Notes: Data from a knowledge, attitudes, and behaviors survey of Medicaid providers in Missouri who prescribed atypical antipsychotic medication in 2013. Missing responses have been excluded (max = 10%). The first 300 surveys were received by day 12.

* Net Promoter Score indicates the likelihood of recommending metabolic screening behaviors to a colleague (0 = “not at all likely”, 10 = “extremely likely”). Detractor = percent reporting 0–6. Neutral = percent reporting 7 or 8. Promoter = percent reporting 9 or 10.

a Pearson’s chi-square test of association *p* < 0.05: First 300 Respondents vs. Later. Highlighted in bold.

b Pearson’s chi-square test of association *p* < 0.05: Within 8 weeks vs. Later. Highlighted in bold.

## Discussion

Evidence-based regulatory decision making, especially considering the role of clinicians and other stakeholders, is an area of proposed regulatory science research and competency development.^27^ In its draft guidance to industry on *REMS Assessment: Planning and Reporting*,^11^ the FDA encourages the research community to develop robust methods for assessing REMS to help advance the science of post-market assessment of the effectiveness of risk mitigation strategies. The European Union (EU) Risk Management Plan (EU-RMP) also requires the assessment of the effectiveness of risk minimization measures (known as RMMs); and assessment has been recognized as an essential element by the European Medicine Agency’s Pharmacovigilance Risk Assessment Committee (PRAC) to inform regulatory actions and to contribute to a proactive EU pharmacovigilance system.^28^^–^^30^ Physician KAB surveys play an important evidentiary role in post-market assessment of drug risk communication and REMS programs.

A key methodologic concern is survey response rate and increased potential for self-selection and biased results. The broader survey research literature has examined numerous factors in response rates. For example, one study tested a set of methodological factors in survey response and found that most efforts to improve response had some effect (with the notable exception of colored paper) and that all the tested methods for improving response increased the cost of survey implementaiton.^31^ However, there has been little examination of these factors in REMS programs or other physician KAB surveys. The studies that have been conducted, such as the systematic review and reporting analysis from Bennett and colleagues,^32^ have identified substantial shortfalls in survey reporting in medical journals.^33^

Our systematic review of 75 physician surveys concerning pharmaceutical use published in the medical literature over a 15-year period found that half of the surveys reported response rates between 34% and 68%. Only one-third of the surveys reviewed (27 out of 75) met the 60% response rate threshold provided in some editorial guidance to authors.^26^ This is in contrast to a review by McLeod an colleagues which examined 117 large (*n* > 500) surveys published in medical journals from 2000 to 2010 and found that 53% of surveys reported a response rate greater than 60%; however, their sample of surveys included surveys of non-providers, which could account for the difference.^33^ Importantly, they also noted a decline in response rates over time, supporting the need for improved survey administration methods and reporting to address nonresponse biases. Physician response rates varied by the size and type of physician population invited to participate and by contact modality. Using multiple (mixed) modalities to contact potential respondents was marginally associated with an average absolute increase in survey response of 14%. Contacting a smaller (more targeted) population of physicians was associated with an absolute increase in survey response of up to 30%. Higher response was also observed when the full census of physicians was known and the total population could be surveyed compared to a random sample of physicians, although this difference was marginally statistically significant.

These findings suggest that to maximize response rates when assessing issues of pharmaceutical use and risk management, physician surveys may be able to achieve higher response when fielded among smaller, targeted populations for whom members are known using a population-based (census) approach and should utilize mixed modalities for contacting physicians. In pragmatic terms, this suggests that conducting several targeted surveys among physicians in different health systems or professional associations might be preferable to conducting a single large representative survey among a national random sample of physicians. Internet-only or mail-only surveys should be avoided. These findings are consistent with survey best practices that state multiple methods of contact, repeated outreach, and intentional sampling can improve response rates and reduce sampling bias.^20^^,^^36^ FDA’s new draft guidance on *Survey Methodologies* also recommends consideration of surveying target populations using probability random sample and using multiple survey administration modalities.^11^

Limitations to this systematic review should be noted. First, many of the survey methodology factors we sought to collect in the reviewed surveys were not reported by the authors, leaving us with substantial missing data for some of the measures. This provides evidence of the need for greater adherence to more comprehensive reporting standards for surveys^37^ and further supports evidence of insufficient survey reporting found by other reviews of the available guidance and medical journal publications.^32^^,^^33^ Second, publication bias may have affected the sample of articles reviewed as studies may be rejected for publication if response did not meet a threshold. In this scenario, surveys with lower response rates would be under-represented and our findings would over-estimate average response rates. For example, 25 articles of the original 175 screened (14%) did not report survey modality, which is a problem itself when striving for data transparency and reproducibility, and had to be excluded from review. In addition, our finding that an internal membership list may be a sample source associated with higher response should be interpreted cautiously in light of half of the reviewed published surveys did not report this information. In addition, best practices in survey methods suggest the importance of monetary incentives for achieving high response;^20^^,^^34^^,^^36^ however, our review suggests that this measure is not reported or is being only sporadically implemented for surveys of physicians (only 23% of studies reported using monetary incentives), making it difficult to assess this as an effective physician survey method for improving response. It should also be noted that the review focused on survey methods and administration and did not examine source of funding, which may affect physician survey response. Lastly, the systematic review may have limited generalizability for physician surveys conducted outside of the USA; however, the response rates reported here are consistent with the range of rates for surveys reported in Europe^37^^,^^38^ and Canada.^39^^,^^40^

As Asch and colleagues have asserted “a survey’s response rate is at best an indirect indication of the extent of non-respondent bias. Investigators, journal editors, and readers should devote more attention to assessments of bias, and less to specific response rate threshholds.”^15^ Probability random sampling design does not protect against nonresponse bias; however, when a bias exists because of nonresponse, probability random sampling gives the ability to quantify the magnitude of this bias to assess its impact on the main findings.^41^ Therefore, it was strikingly noteworthy to find that none of the physician surveys examined in this literature review provided an assessment of nonrespondent bias, either in the text or in supplemental materials. This is a standard survey reporting practice across disciplines^32^^,^^42^^–^^45^ and should be part of standard regulatory reporting for REMS and other pharmaceutical risk management surveys.

In the second part of this mixed-methods evaluation, we sought to evaluate response bias and compare survey results using different strategies for determining when to close enrollment and end the survey. This study design consideration has important implications in the context of regulatory decision-making and significant pressure for timeliness of results. We examined two early close strategies – (a) reach a prespecified sample (*n* = 300) or (b) reach a prespecified end date (8 weeks) – versus using the scientific strategy to maximize survey response until the survey reaches futility in enrollment (e.g., 6 months in the present case). A strength of our evaluation was that we were able to simulate responders and nonresponders and compare their characteristics and responses using a case study simulation within a large population-based survey.

Our analysis found fewer statistically significant differences between respondent and simulated nonrespondent groups on knowledge and attitudinal statements using the 8-week endpoint (1 out of 12 [8%] survey items) than using the fixed sample endpoint (*n* = 300) common in polling survey designs (2 out of 20 [17%] items). The difference was even more striking when examining the survey items related to risk management behaviors. Using an 8-week endpoint resulted in only one of eight of the behavioral survey items being significantly different in responders versus simulated nonresponders; whereas, using the fixed sample approach resulted in 7 survey items out of 8 being different. These differences may be related to the finding that the earliest responders to this physician survey were characteristically different in important ways. For example, the threshold of 300 respondents was achieved in less than 2 weeks, and these very early survey responders had lower rates of antipsychotic prescribing and were less likely to treat patients in community-mental health centers (which represents 24.3% of new starts).^46^

REMS evaluation requires timely evidence for regulatory decision making. Findings from a simulation study of secondary data from a population-based survey using a probabilistic sampling frame suggest that an 8-week endpoint combined with rigorous, multimodal outreach may reduce the potential for respondent-bias while balancing the need for rapid survey data collection. The simulation findings suggest that using a predetermined time period, for example, 8 weeks, to administer population-based physician surveys using probability sampling may be preferable than using a predetermined sample size. The caveat is that survey administration should be robust and include multiple modes of contact and multiple outreach attempts during that time period (three or more), as was done in the source survey. After 8 weeks there were diminishing returns in survey response, and the yield from additional outreach attempts was small. Thus, 8 weeks appears to represent a pragmatic approach in maximizing response and representativeness of the sample while ensuring timely results for regulatory decision making. Eight weeks has also been the standard used in physician surveying to rapidly investigate emerging vaccine policy-relevant policy making by the Centers for Disease Control and Prevention.^19^^,^^47^^–^^50^

The simulation findings also show the value of graphing response curves over time to visually monitor study progress and assess potential for respondent bias. This is akin to tracking patient accrual rates into a clinical trial over time to assess screening yield and generalizability of the study population. In the antipsychotic population-based survey examined, it is evident from the graph that a fixed sample approach rapidly filled the quota but only captured the tip of early respondents. Sometimes pharmaceutical risk management surveys have difficulty even achieving the fixed sample size target. In this case, graphing response over time can be used to investigate the return on yield from waves of outreach attempts and when response has plateaued. Graphing total response rates (completed surveys plus active opt-outs) also showed greater representation and reach of the survey; a segment of physicians had engaged and concluded they did not meet eligibility criteria.

The case study simulation was conducted using a dataset for a population-based survey conducted among physicians prescribing antipsychotic medication within Missouri Medicaid. It is unknown whether the findings would apply to other physician surveys on other types of medications or in other healthcare settings. Physicians seeing a larger proportion of patients who have Medicaid and may also have both more risk factors for diabetes and access to healthcare may have more knowledge of risk mitigation and may also be more or less likely to respond to surveys than those who do not treat Medicaid patients. More research is needed to investigate and replicate these findings in other state and other provider populations. Our goal was to provide a structured means for examining the impact of survey design considerations in pharmaceutical risk management surveys to inform survey design and reporting standards.

**Implications:** Using rigorous survey methodology, including allowing sufficient time to collect physician survey responses, does not remove all sources of potential bias from physician KAB surveys, but it can reduce differences between responders and nonresponders on key, clinically significant measures. The goal for this mixed-methods evaluation was to advance regulatory science and the assessment of FDA REMS programs when using physician surveys. Formal analyses of survey responder bias should be performed for physician surveys used to assess the effectiveness of REMS programs and other pharmaceutical risk management interventions. Increasing requirements for response rate reporting, response bias analysis, and survey method choices for conducting KAB surveys as part of REMS could improve the ability of the FDA and manufacturers to accurately assess and, thereby, more effectively mitigate risk associated with medications.

## Supplementary material

For supplementary material accompanying this paper visit https://doi.org/10.1017/cts.2019.400. click here to view supplementary material
