# Supplementary material for: Advancing regulatory science and assessment of FDA REMS programs: A mixed-methods evaluation examining physician survey response
Source: J Clin Transl Sci. 2019 Sep 13;3(4):199–209. doi: 10.1017/cts.2019.400 (PMC6799639; doi:10.1017/cts.2019.400)
Supplement: Supplementary file 1 [file S205986611900400Xsup.zip › S205986611900400Xsup001.docx]

**Supplementary Text: Full citations for included surveys**

Abourjaily P, Gouveia WA, Selker HP, Zucker DR. Evaluating the nondrug costs of formulary coverage restrictions. *Manag Care.* 2005;14(8):50-57, 62.

Alkhateeb FM, Unni E, Latif D, Shawaqfeh MS, Al-Rousan RM. Physician attitudes toward collaborative agreements with pharmacists and their expectations of community pharmacists' responsibilities in West Virginia. *J Am Pharm Assoc (2003).* 2009;49(6):797-800.

Audet AM, Doty MM, Peugh J, Shamasdin J, Zapert K, Schoenbaum S. Information technologies: when will they make it into physicians' black bags? *MedGenMed.* 2004;6(4):2.

Barrett K, Watson A. Physician perspectives on a pilot prescription monitoring program. *J Pain Palliat Care Pharmacother.* 2005;19(3):5-13.

Bellanger RA, Shank TC. Continuing professional development in Texas: survey of pharmacists' knowledge and attitudes: 2008. *J Am Pharm Assoc (2003).* 2010;50(3):368-374.

Blake EW, Blair MM, Couchenour RL. Perceptions of pharmacists as providers of immunizations for adult patients. *Pharmacotherapy.* 2003;23(2):248-254.

Blendon RJ, DesRoches CM, Brodie M, et al. Views of practicing physicians and the public on medical errors. *New England Journal of Medicine.* 2002;347(24):1933-1940.

Buckley PF, Miller DD, Singer B, Arena J, Stirewalt EM. Clinicians' recognition of the metabolic adverse effects of antipsychotic medications. *Schizophr Res.* 2005;79(2-3):281-288.

Bujold E, Huff J, Staton EW, Pace WD. Improving use of narcotics for nonmalignant chronic pain: a lesson from Community Care of North Carolina. *Journal of opioid management.* 2012;8(6):363-367.

Burke R, Spoerri M, Price A, Cardosi A-M, Flanagan P. Survey of primary care pediatricians on the transition and transfer of adolescents to adult health care. *Clin Pediatr (Phila).* 2008;47(4):347-354.

Carayon P, Hundt AS, Alvarado C, Springman S, Ayoub P. Patient safety in outpatient surgery: the viewpoint of the healthcare providers. *Ergonomics.* 2006;49(5-6):470-485.

Carroll AE, Christakis DA. Pediatricians’ use of and attitudes about personal digital assistants. *Pediatrics.* 2004;113(2):238-242.

Charuvastra A, Friedmann PD, Stein MD. Physician attitudes regarding the prescription of medical marijuana. *J Addict Dis.* 2005;24(3):87-93.

Chen DT, Wynia MK, Moloney RM, Alexander GC. U.S. physician knowledge of the FDA-approved indications and evidence base for commonly prescribed drugs: results of a national survey. *Pharmacoepidemiol Drug Saf.* 2009;18(11):1094-1100.

Copher R, Buzinec P, Zarotsky V, Kazis L, Iqbal SU, Macarios D. Physician perception of patient adherence compared to patient adherence of osteoporosis medications from pharmacy claims. *Curr Med Res Opin.* 2010;26(4):777-785.

Craig A, Cronin B, Eward W, et al. Attitudes toward physician-assisted suicide among physicians in Vermont. *J Med Ethics.* 2007;33(7):400-403.

Crane LA, Daley MF, Barrow J, et al. Sentinel physician networks as a technique for rapid immunization policy surveys. *Eval Health Prof.* 2008;31(1):43-64.

Danhauer JL, Johnson CE, Rotan SN, Snelson TA, Stockwell JS. National survey of pediatricians' opinions about and practices for acute otitis media and xylitol use. *J Am Acad Audiol.* 2010;21(5):329-346.

Dhanda Patil R, Patil YJ. Perioperative management of obstructive sleep apnea: a survey of Veterans Affairs health care providers. *Otolaryngology--Head and Neck Surgery.* 2012;146(1):156-161.

Doron S, Nadkarni L, Lyn Price L, et al. A nationwide survey of antimicrobial stewardship practices. *Clin Ther.* 2013;35(6):758-765.e720.

Ernst ME, Bergus GR, Sorofman BA. Patients' acceptance of traditional and nontraditional immunization providers. *J Am Pharm Assoc (Wash).* 2001;41(1):53-59.

Evans RW, Lipton RB, Ritz KA. A survey of neurologists on self-treatment and treatment of their families. *Headache.* 2007;47(1):58-64.

Fagan MJ, Chen JT, Diaz JA, Reinert SE, Stein MD. Do internal medicine residents find pain medication agreements useful? *Clin J Pain.* 2008;24(1):35-38.

Fjortoft N, Zgarrick D. An assessment of pharmacists' caring ability. *J Am Pharm Assoc (2003).* 2003;43(4):483-487.

Freed GL, Dunham KM, Lamarand KE, Loveland-Cherry C, Martyn KK, Committee ABoPRA. Pediatric nurse practitioners: roles and scope of practice. *Pediatrics.* 2010:peds. 2010-1589.

Glassman PA, Good CB, Kelley ME, Bradley M, Valentino M. Physician satisfaction with formulary policies: Is it access to formulary or nonformulary drugs that matters most? *Am J Manag Care.* 2004;10(3):209-216.

Glassman PA, Tanielian T, Harris K, et al. Provider perceptions of pharmacy management: lessons from the military health system. *Med Care.* 2004;42(4):361-366.

Gross CP, Vogel EW, Dhond AJ, et al. Factors influencing physicians' reported use of anticoagulation therapy in nonvalvular atrial fibrillation: a cross-sectional survey. *Clin Ther.* 2003;25(6):1750-1764.

Hermes ED, Sernyak M, Rosenheck R. Use of second-generation antipsychotic agents for sleep and sedation: a provider survey. *Sleep.* 2013;36(4):597-600.

Huang C, Siu M, Vu L, Wong S, Shin J. Factors influencing doctors' selection of dabigatran in non-valvular atrial fibrillation. *J Eval Clin Pract.* 2013;19(5):938-943.

Hughes EG, DeJean D. Cross-border fertility services in North America: a survey of Canadian and American providers. *Fertility and Sterility.* 2010;94(1):e16-e19.

Hurley LP, Lindley MC, Harpaz R, et al. Barriers to the use of herpes zoster vaccine. *Ann Intern Med.* 2010;152(9):555-560.

Hyman DJ, Pavlik VN. Characteristics of patients with uncontrolled hypertension in the United States. *New England Journal of Medicine.* 2001;345(7):479-486.

Jepson C, Asch DA, Hershey JC, Ubel PA. In a mailed physician survey, questionnaire length had a threshold effect on response rate. *Journal of clinical epidemiology.* 2005;58(1):103-105.

Khan S, Sylvester R, Scott D, Pitts B. Physicians' opinions about responsibility for patient out-of-pocket costs and formulary prescribing in two Midwestern states. *J Manag Care Pharm.* 2008;14(8):780-789.

Kucukarslan S, Lai S, Dong Y, Al-Bassam N, Kim K. Physician beliefs and attitudes toward collaboration with community pharmacists. *Res Social Adm Pharm.* 2011;7(3):224-232.

McFarlane E, Olmsted MG, Murphy J, Hill CA. Nonresponse bias in a mail survey of physicians. *Eval Health Prof.* 2007;30(2):170-185.

McMahon SR, Iwamoto M, Massoudi MS, et al. Comparison of e-mail, fax, and postal surveys of pediatricians. *Pediatrics.* 2003;111(4):e299-e303.

McMahon SR, Iwamoto M, Massoudi MS, et al. Comparison of e-mail, fax, and postal surveys of pediatricians. *Pediatrics.* 2003;111(4):e299-e303.

Menachemi N, Lee SC, Shepherd JE, Brooks RG. Proliferation of electronic health records among obstetrician-gynecologists. *Qual Manag Health Care.* 2006;15(3):150-156.

Newcomer JW, Nasrallah HA, Loebel AD. The atypical antipsychotic therapy and metabolic issues national survey: practice patterns and knowledge of psychiatrists. *J Clin Psychopharmacol.* 2004;24(5):S1-S6.

Newton KM, LaCroix AZ, Buist DS, Anderson LA, Delaney K. What factors account for hormone replacement therapy prescribing frequency? *Maturitas.* 2001;39(1):1-10.

Nichol KL, Zimmerman R. Generalist and subspecialist physicians' knowledge, attitudes, and practices regarding influenza and pneumococcal vaccinations for elderly and other high-risk patients: a nationwide survey. *Archives of internal medicine.* 2001;161(22):2702-2708.

Nutescu EA, Pickard AS, Blackburn JC, Wittkowsky AK, Ansell J, Schumock GT. Impact of oral direct thrombin inhibitors on anticoagulation clinics. *Pharmacotherapy.* 2004;24(9):1204-1212.

Pedersen CA, Schneider PJ, Scheckelhoff DJ. ASHP national survey of pharmacy practice in hospital settings: prescribing and transcribing--2004. *Am J Health Syst Pharm.* 2005;62(4):378-390.

Pedersen CA, Schneider PJ, Scheckelhoff DJ. ASHP national survey of pharmacy practice in hospital settings: Monitoring and patient education--2009. *Am J Health Syst Pharm.* 2010;67(7):542-558.

Pinto SL, Lipowski E, Segal R, Kimberlin C, Algina J. Physicians' intent to comply with the American Medical Association's guidelines on gifts from the pharmaceutical industry. *J Med Ethics.* 2007;33(6):313-319.

Ponte CD, Johnson-Tribino J. Attitudes and knowledge about pain: an assessment of West Virginia family physicians. *Fam Med.* 2005;37(7):477-480.

Rebuck JA, Rasmussen JR, Olsen KM. Clinical aspiration-related practice patterns in the intensive care unit: a physician survey. *Crit Care Med.* 2001;29(12):2239-2244.

Reichert S, Simon T, Halm EA. Physicians' attitudes about prescribing and knowledge of the costs of common medications. *Arch Intern Med.* 2000;160(18):2799-2803.

Rhoney DH, Murry KR. National survey of the use of sedating drugs, neuromuscular blocking agents, and reversal agents in the intensive care unit. *J Intensive Care Med.* 2003;18(3):139-145.

Rich JD, Whitlock TL, Towe CW, et al. Prescribing syringes to prevent HIV: a survey of infectious disease and addiction medicine physicians in Rhode Island. *Subst Use Misuse.* 2001;36(5):535-550.

Rushton JL, Clark SJ, Freed GL. Pediatrician and family physician prescription of selective serotonin reuptake inhibitors. *Pediatrics.* 2000;105(6):E82.

Schaffer SJ, Humiston SG, Shone LP, Averhoff FM, Szilagyi PG. Adolescent immunization practices: a national survey of US physicians. *Archives of pediatrics & adolescent medicine.* 2001;155(5):566-571.

Schmuhl P, Van Duker H, Gurley KL, Webster A, Olson LM. Reaching emergency medical services providers: is one survey mode better than another? *Prehosp Emerg Care.* 2010;14(3):361-369.

Seib K, Gleason C, Richards JL, et al. Partners in immunization: 2010 survey examining differences among H1N1 vaccine providers in Washington state. *Public Health Rep.* 2013;128(3):198-211.

Shaffer EG, Moss AH. Physicians' perceptions of doctor shopping in West Virginia. *W V Med J.* 2010;106(4 Spec No):10-14.

Shrank WH, Young HN, Ettner SL, Glassman P, Asch SM, Kravitz RL. Do the incentives in 3-tier pharmaceutical benefit plans operate as intended? Results from a physician leadership survey. *Am J Manag Care.* 2005;11(1):16-22.

Simon SR, Kaushal R, Cleary PD, et al. Physicians and electronic health records: a statewide survey. *Arch Intern Med.* 2007;167(5):507-512.

Sinkowitz-Cochran RL, Stein GP, Keyserling HL, Levine GL, Jarvis WR. The Internet: a practical example of the use of new technology in the assessment of vancomycin use in pediatrics. The Pediatric Prevention Network. *Am J Infect Control.* 2000;28(6):459-464.

Sleath BL, Thomas N, Jackson E, West SL, Gaynes BN. Physician reported communication about depression and psychosocial issues during postpartum visits. *N C Med J.* 2007;68(3):151-155.

Sohn W, Ismail AI, Taichman LS. Caries risk-based fluoride supplementation for children. *Pediatr Dent.* 2007;29(1):23-31.

Sox CM, Christakis DA. Pediatricians' screening urinalysis practices. *The Journal of pediatrics.* 2005;147(3):362-365.

Spina JR, Glassman PA, Simon B, et al. Potential safety gaps in order entry and automated drug alerts: a nationwide survey of VA physician self-reported practices with computerized order entry. *Med Care.* 2011;49(10):904-910.

Wall GC, Dewitt JE, Haack S, Fornoff A, Eastman DK, Koenigsfeld CF. Knowledge and attitudes of American pharmacists concerning sulfonamide allergy cross-reactivity. *Pharm World Sci.* 2010;32(3):343-346.

Wilson JE, Kiselanova N, Stevens Q, et al. A survey of inhalational anaesthetic abuse in anaesthesia training programmes. *Anaesthesia.* 2008;63(6):616-620.

Wischmeyer PE, Johnson BR, Wilson JE, et al. A survey of propofol abuse in academic anesthesia programs. *Anesth Analg.* 2007;105(4):1066-1071, table of contents.

Wolfert MZ, Gilson AM, Dahl JL, Cleary JF. Opioid analgesics for pain control: wisconsin physicians' knowledge, beliefs, attitudes, and prescribing practices. *Pain Med.* 2010;11(3):425-434.

Xu X, Vahratian A, Patel DA, McRee AL, Ransom SB. Emergency contraception provision: a survey of Michigan physicians from five medical specialties. *Journal of women's health (2002).* 2007;16(4):489-498.

Zachry WM, 3rd, Dalen JE, Jackson TR. Clinicians' responses to direct-to-consumer advertising of prescription medications. *Arch Intern Med.* 2003;163(15):1808-1812.
