# Supplementary material for: Advancing regulatory science and assessment of FDA REMS programs: A mixed-methods evaluation examining physician survey response
Source: J Clin Transl Sci. 2019 Sep 13;3(4):199–209. doi: 10.1017/cts.2019.400 (PMC6799639; doi:10.1017/cts.2019.400)
Supplement: Supplementary file 1 [file S205986611900400Xsup.zip › S205986611900400Xsup002.docx]

**Supplementary Figure 1 – CONSORT Diagram of search and review.**


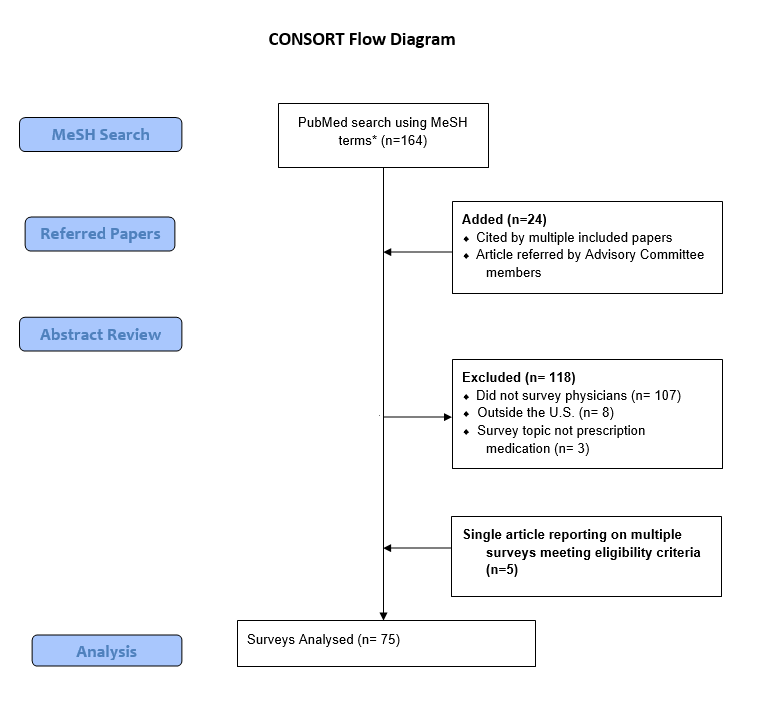


***Note:** MeSH and keyword search terms: (((((((((((((("Health Care Surveys"[Mesh] OR "Health Surveys"[Mesh] OR "Data Collection"[Mesh] OR Nonrespondents OR Nonrespondent OR Respondents OR Respondent OR Surveys OR Survey OR "Questionnaires"[Mesh] OR "Questionnaires" OR Questionnaire))))))

AND

((("Physicians"[Mesh] OR physicians OR physician OR "Health Personnel"[Mesh] OR "Health Care Providers" OR "Health Care Provider" OR "Healthcare Providers" OR "Healthcare Provider"))))

AND

((("response rate" OR "response rates"))))

AND

((("United States"[Mesh] OR "United States")))))

AND

("Pharmacy"[Mesh] OR "Pharmacy" OR OR "Prescriptions"[Mesh] OR "Prescriptions")))

AND

( "2000/01/01"[PDat] : "2014/12/31"[PDat] ) AND English[lang]))

AND

(United States[ad] OR US[ad] OR America[ad] OR USA[ad])
