# Supplementary material for: Advancing regulatory science and assessment of FDA REMS programs: A mixed-methods evaluation examining physician survey response
Source: J Clin Transl Sci. 2019 Sep 13;3(4):199–209. doi: 10.1017/cts.2019.400 (PMC6799639; doi:10.1017/cts.2019.400)
Supplement: Supplementary file 1 [file S205986611900400Xsup.zip › S205986611900400Xsup003.docx]

**Appendix B – Part 1: Characteristics of Articles Meeting Review Eligibility**

| **Pub Year** | **1st Author** | **Response Rate (%)** | **Location** | **Sample Source^a^** | **Sampling Method^b^** | **Number Contacted** | **Contact Method** | **Contact Attempts** | **Incentive Amount^c^** |
| --- | --- | --- | --- | --- | --- | --- | --- | --- | --- |
| 2000 | Hyman & Pavlik | 34 | National | Commercial | Random Sample | 1,200 | Mixed | 5 | NR |
| 2000 | Sinkowitz-Cochran, et al. | 63 | National | NR | Population | 54 | Email/Online | NR | NR |
| 2000 | Rushton, et al. | 66 | State/Local | NR | Population, Random | 1,016 | Mail | 3 | NR |
| 2000 | Reichert, et al. | 71 | State/Local | Internal | Population | 189 | Mail | 2 | NR |
| 2001 | Rebuck, et al. | 32 | National | NR | Random Sample | 2,000 | Mail | 3 | NR |
| 2001 | Nichol & Zimmerman | 32 | National | Commercial | Random Sample | 6,000 | Mail | 3 | NR |
| 2001 | Ernst | 67 | State/Local | NR | Population | 627 | NR | NR | NR |
| 2001 | Shaffer, et.al. | 75 | National | Commercial | Random Sample | 1,480 | Mixed | 5 | NR |
| 2001 | Rich, et al. | 80 | State/Local | NR | Population | 49 | Mixed | 3 | NR |
| 2001 | Newton, et al. | 83 | State/Local | Internal | Population | 480 | Mail | 3 | $25 |
| 2002 | Blendon, et.al. | 62 | National | Commercial | Random Sample | 1,332 | Mixed | 1 | $100 |
| 2003 | Fjortoft, et al. | 30 | State/Local | NR | Random Sample | 1,076 | Mail | NR | NR |
| 2003 | Gross, et.al | 33 | National | Commercial | Random Sample | 426 | Mixed | 3 | $25 |
| 2003 | Zachry, et al. | 44 | State/Local | NR | Population | 1,784 | Mail | 1 | NR |
| 2003 | Rhoney, Murry | 50 | National | NR | Population | 1,934 | Mail | 1 | NR |
| 2003 | McMahon, et.al. | 53 | State/Local | Internal | RCT- 3 Arms | 450 | Mixed | 3 | NR |
| 2004 | Newcomer, et.al. | 30 | National | Commercial | Poll | 1,000 | Other | NR | $75 |
| 2004 | Nutescu, et al. | 35 | National | NR | Random Sample | 400 | Mail | 3 | NR |
| 2004 | Glassman, et al. | 38 | National | Internal | Random Sample | 557 | Mixed | 5 | 0 |
| 2004 | Audet, et al. | 53 | National | Commercial | Random Sample | 3,479 | Mixed | NR | NR |
| 2004 | Delnevo | 56 | State/Local | Commercial | Random Sample | 2,100 | Mail | 3 | $25 |
| 2004 | Carroll & Chritakis | 63 | National | Commercial | Random Sample | 2,130 | Mail | 3 | $1 |
| 2004 | Glassman, et al. | 69 | National | NR | Random Sample | 566 | Mixed | 5 | NR |
| 2004 | Delnevo | 72 | State/Local | Commercial | Random Sample | 2,100 | Mail | 3 | $25 |
| 2005 | Jepson† | 17 | National | Commercial | Random Sample | 1,700 | Mail | 1 | NR |
| 2005 | Buckley, et.al. | 17 | State/Local | NR | Population | 1,534 | Mail | 1 | NR |
| 2005 | Abourjaily, et al. | 18 | National | Internal | Population | 569 | Email/Online | 4 | NR |
| 2005 | Ponte, et al. | 35 | State/Local | NR | Population | 537 | Mail | 1 | NR |
| 2005 | Barrett, et al. | 41 | State/Local | NR | Population | 672 | Mixed | 2 | NR |
| 2005 | Sox & Christakis | 50 | National | Commercial | Random Sample | 1,502 | Mail | 4 | $1 |
| 2005 | Jepson† | 60 | National | Commercial | Random Sample | 1,700 | Mail | 1 | NR |
| 2005 | Shrank, et al. | 65 | National | NR | Convenience Sample | 205 | Other | 1 | NR |
| 2005 | Charuvastra, et al. | 66 | National | Commercial | National Systematic Sample | 2,227 | Mail | 3 | NR |
| 2006 | Menachemi, et al. | 28 | State/Local | NR | Random Sample | 14,192 | Mail | 2 | NR |
| 2006 | Pedersen, et al. | 44 | National | NR | Random Sample | 1,173 | Mail | 6 | $25 |
| 2006 | Carayon | 53 | State/Local | NR | Population | 531 | Other | 1 | NR |
| 2007 | Pinto, et al. | 26 | State/Local | NR | Population | 850 | Mixed | 3 | NR |
| 2007 | Xu, et al. | 32 | State/Local | NR | Random Sample | 850 | Mail | 2 | NR |
| 2007 | Sleath, et al. | 42 | State/Local | NR | Random Sample | 1,200 | Mail | 2 | NR |
| 2007 | Sohn, et al. | 43 | National | NR | Representative Sample |  | Mail | NR | NR |
| 2007 | McFarlane, et.al. | 47 | National | Commercial | Random sample | 3,400 | Mail | 4 | $2 |
| 2007 | Evans, et al. | 48 | National | NR | Population | 186 | Other | 1 | NR |
| 2007 | Craig, et al. | 48 | State/Local | NR | Population | 2,770 | Mail | 3 | NR |
| 2007 | Simon, et al. | 71 | State/Local | NR | Random Sample | 1,884 | Mixed | 4 | $20 |
| 2007 | Wischmeyer, et al. | 100 | National | Internal | Selection from Pop | 23 | Mixed | NR | NR |
| 2007 | Wischmeyer, et al. | 100 | National | Internal | Population | 126 | Mixed | 4 | NR |
| 2008 | Crane, et.al. | 36 | National | Commercial | Random Sample | 1,000 | Mail | 3 | NR |
| 2008 | Khan, et al. | 50 | State/Local | NR | Random Sample | 690 | Mixed | 6 | NR |
| 2008 | Burke, et al. | 61 | State/Local | NR | Population | 169 | Mixed | 4 | NR |
| 2008 | Crane, et.al. | 76 | National | Commercial | Pre-recruited Panel | 1,357 | Mail | 3 | NR |
| 2008 | Wilson, et al. | 84 | National | NR | Population | 126 | Mixed | 4 | NR |
| 2008 | Fagan, et al. | 89 | State/Local | Internal | Population | 124 | NR | NR | NR |
| 2009 | Alkhateeb, et al. | 22 | State/Local | Commercial | Random Sample | 500 | Mail | 3 | Drawing |
| 2009 | Chen, et al. | 47 | National | Commercial | Random Sample | 1,199 | Mail | 5 | NR |
| 2010 | Bellanger, Shank | 10 | State/Local | Commercial | Population | 4,954 | Email | 2 | NR |
| 2010 | Copher, et al. | 22 | National | Commercial | Population (Docs PMO patients) | 2,000 | Mail | 1 | NR |
| 2010 | Danhauer, et al. | 22 | National | NR | Random Sample | 506 | Mail | 1 | NR |
| 2010 | Hughes, et al. | 32 | National | NR | Population | 392 | Email/Online | 2 | NR |
| 2010 | Schmuhl | 36 | National | NR | RCT - 3 arms | 1,873 | Mail | 3 | $1 |
| 2010 | Wolfert, et al. | 36 | State/Local | NR | Random Sample | 600 | Mixed | 3 | $2 |
| 2010 | Wall, et al. | 39 | State/Local | NR | Population | 1,086 | Mixed | NR | 0 |
| 2010 | Pedersen, et al. | 41 | National | Internal | Random Sample | 1,364 | Mail | NR | NR |
| 2010 | Shaffer, Moss | 57 | State/Local | NR | Random Sample | 452 | Mixed | 2 | NR |
| 2010 | Hurley, et al. | 72 | National | NR | Quota Sampling | 828 | Mixed | 11 | NR |
| 2010 | Freed, et.al | 82 | National | Commercial | Random Sample | 1,200 | Mail | 3 | $5 |
| 2011 | Kucukarslan, et al. | 30 | State/Local | Commercial | Random Sample | 1,206 | Mail | 4 | $5 |
| 2011 | Spina, et al. | 43 | National | Internal | Random Selection | 3,588 | Mixed | 5 | NR |
| 2012 | Blake, et al. | 48 | State/Local | NR | Convenience Sample | 131 | Other | 2 | NR |
| 2012 | Blake, et al. | 71 | State/Local | Internal | Population | 28 | Mail | 1 | NR |
| 2012 | Bujold, et al. | 77 | State/Local | NR | Population | 35 | Other | NR | NR |
| 2012 | Patil & Patil | 80 | System | Internal | Population | 128 | Email/Online | NR | Mandated |
| 2012 | Hermes, et.al. | 83 | System | Internal | Population | 2,643 | Other | NR | Mandated |
| 2013 | Doron, et al. | 7 | National | Commercial | Targeted Selection | 5,890 | Email/Online | 4 | NR |
| 2013 | Huang, et al. | 36 | State/Local | Internal | Population | 181 | Mixed | 5 | NR |
| 2013 | Seib, et al. | 81 | State/Local | NR | Random Sample | 800 | Mixed | 7 | $25 |

NR = not reported; RCT = randomized controlled trial

**^a^** Sample source indicates whether original contact list came from an internal membership list (“Internal”) or a purchased commercial source (“Commercial”).

**^b^** Sampling methods describes how the sample was selected from the sample source list: “Population” = all members contacted; “Targeted selection” = sample selected by defined criteria; “Convenience sample” = sample drawn by ease to reach individual; “Random sample” (or “RCT”) = sample was drawn randomly from full population; “Pre-recruited Panel” = sample was recruited prior to survey recruitment; “Poll” = total population invited and enrollment closed after pre-defined number of responses; “Representative sample” = sample drawn based on demographic factors; “Quota sampling” = sample was drawn to meet minimum number of responses on certain demographic factors.

^c “^ “Mandated” = participants were required to participate as part of membership or employment; “Drawing” = participants were entered in a drawing for a limited number of incentives/awards;
